# Novel high strength PVA/soy protein isolate composite hydrogels and their properties

**DOI:** 10.3389/fchem.2022.984652

**Published:** 2022-08-22

**Authors:** Yanteng Zhao, Lu Jin, Xin Liu, Xue Liu, Shuling Dong, Yun Chen, Xianyu Li, Xianping Lv, Meng He

**Affiliations:** ^1^ Department of Transfusion, The First Affiliated Hospital of Zhengzhou University, Zhengzhou, China; ^2^ Department of Biomedical Engineering and Hubei Province Key Laboratory of Allergy and Immune Related Diseases, School of Basic Medical Sciences, Wuhan University, Wuhan, China; ^3^ School of Materials Science and Engineering, Yancheng Institute of Technology, Yancheng, China; ^4^ Department of Pathophysiology, School of Basic Medical Sciences, Hubei University of Medicine, Shiyan, China

**Keywords:** high strength hydrogel, soy protein isolate, polyvinyl alcohol, biocompatibility, hemocompatibility

## Abstract

High strength polyvinyl alcohol (PVA)/soy protein isolate (SPI) composite hydrogels (EPSG) were constructed by the introduction of PVA into SPI through the crosslinking with epichlorohydrin (ECH) and a freezing-thawing process. The EPSG hydrogels were characterized by scanning electron microscopy, FTIR, X-ray diffraction and compressive test. The results revealed that chemical crosslinking interactions occurred for SPI and PVA during the fabrication process. The composite hydrogels exhibited a homogenous porous structure, indicating certain miscibility between PVA and SPI. The introduction of PVA increased the compressive strength of SPI hydrogels greatly, which could reach as high as 5.38 MPa with the water content ratio of 89.5%. Moreover, the water uptake ratio of completely dried SPI hydrogel (namely xerogel) decreased gradually from 327.4% to 148.1% with the incorporation of PVA, showing a better potential as implants. The cytocompatibility and hemocompatibility of the EPSG hydrogels were evaluated by a series of *in vitro* experiments. The results showed that the EPSG hydrogels had no cytotoxicity (cell viability values were above 86.7%), good biocompatibility and hemocompatibility, showing potential applications as a direct blood contact material in the field of tissue engineering.

## 1 Introduction

Hydrogels are soft three-dimensional hydrophilic materials that exhibit good flexibility and bio-friendliness, making them widely used in various fields including scaffolds for tissue engineering, carriers for drug delivery and wound dressings etc (Seliktar, 2012; Kumar et al., 2019; Zhao C et al., 2019; Liang et al., 2020; Ai et al., 2021). Recently, the use of biopolymer-based hydrogels has attracted much attention for biomedical applications due to their low toxicity, renewability and biocompatibility (Li C et al., 2020; Ounkaew et al., 2020). As a kind of renewable biopolymer, soy protein isolate (SPI) is the most abundant source of plant protein, which has advantages of abundance, low cost, good biodegradability and biocompatibility, showing an important role in the sustainable production of chemicals and materials (Silva et al., 2014; Ashaolu, 2020). SPI can be processed into films, fibers, sponges, plastics, gels and microspheres, which could be used widely in the fields of drug control release and tissue engineering etc (Tian K et al., 2010; Zhao et al., 2016; Zhao et al., 2018; Zhao Y et al., 2019; Las Heras et al., 2020). However, pure SPI based materials usually have water sensitivity and relatively poor mechanical properties, which prevent them from being used as structural materials in the tissue engineering field (Guo et al., 2015). Pure SPI physical hydrogels could be fabricated physically by a heating or cooling gelation process (Chen and Subirade, 2009). But SPI physical hydrogels usually lack good mechanical property, so chemical crosslinkers such as epichlorohydrin (ECH) could be introduced to improve their water stability and mechanical strength to some extent (Tian K et al., 2010). Moreover, the common practices to resolve the poor mechanical properties of SPI materials mainly include blending with other polymers, and/or reinforcing with fillers (Tian H. et al., 2010; Tian, 2012; Liu et al., 2017).

Among the reinforcing polymers, poly (vinyl alcohol) (PVA) is a biodegradable polymer from a petroleum source, which have advantages including chemical resistance, non-toxicity, water-solubility, biocompatibility and biodegradability as well as good processability (Li P et al., 2020). Elastic and high strength PVA hydrogels can be fabricated facilely by a freezing-thawing process, which could yield small crystalline nuclei through the inter-molecular interaction of PVA (Mori et al., 1997; Hassan and Peppas, 2000; Ai et al., 2021). PVA had been combined with gelatin, agar, chitin, cellulose and chitosan to fabricate composite hydrogels with improved mechanical properties and good biocompatibility (Yang et al., 2008; He et al., 2014; Eivazzadeh-Keihan et al., 2020; Han et al., 2020; Wang et al., 2020; Bai et al., 2021; Geng et al., 2021). Moreover, blending PVA with SPI have been considered a reasonable choice to improve the mechanical properties of SPI materials (Su et al., 2010; Iqbal et al., 2020; Li C et al., 2020; Li et al., 2021), and a series of SPI/PVA composite materials such as fibers, films and plastics with or without crosslinkers have been fabricated (Su et al., 2010; Cho et al., 2012; Guo et al., 2015; Won et al., 2015).

However, as far as we know, high strength SPI/PVA composite hydrogels have never been reported. Thus, a worthwhile endeavor would be to construct novel high strength SPI/PVA composite hydrogels by the combination of the chemical crosslinking and a freezing-thawing process. In the present work, SPI was dissolved in alkaline aqueous solution, and then composite hydrogels were prepared by adding PVA aqueous solution into SPI solution by using ECH as a chemical crosslinker. The freezing-thawing process was then used to promote the formation of PVA crystals to reinforce hydrogels. The structure of the resultant SPI/PVA hydrogels was characterized systematically, and the cytocompatibility and hemocompatibility were also evaluated, hoping to obtain novel high strength biomaterials with good biocompatibility and hemocompatibility on the basis of SPI and PVA.

## 2 Experiment section

### 2.1 Materials

Epichlorohydrin (ECH), PVA (1788), NaOH, CaCl_2_ and acetic acid were obtained from Sinopharm Chemical Reagent Co. Ltd. (Shanghai, China). Soy protein isolate (SPI, Mw of 2.05×10^5^), was purchased from DuPont-Yunmeng Protein Technology. Modified Eagle’s Medium (MEM) and MTT were purchased from Invitrogen Corporation (Gibco BRL, Grand Island, NY, United States). Other chemicals were all analytically pure and used directly without further treatment.

### 2.2 Preparation of PVA/soy protein isolate composite hydrogels

A viscous 10% SPI aqueous solution was prepared by dispersing SPI powder into deionized water to completely swollen and dissolving further by the addition of a 5 wt% NaOH aqueous solution. 10 g PVA was dissolved in 90 g deionized water at 95 °C with mechanical stirring to obtain 10% PVA solutions. The above SPI and PVA solutions were mixed with different weight ratios, then desired amounts of ECH (20% of the total dry weight of the original SPI and PVA) were added in the mixed SPI/PVA solutions. The resultant solutions were poured into square or tubular molds and then frozen at -20 °C in a refrigerator for 12 h with the following thawing process at room temperature. The freezing-thawing process was repeated for four cycles to complete the gelation process. Then, the resultant samples were treated in a 5% acetic acid solution for 4 h and rinsed completely using deionized water. The resultant ECH crosslinked PVA/SPI composite hydrogels were coded as EPSG-n, n corresponds to the initially designed PVA weight content (n = 0, 30, 50, 70 and 100, respectively). The codes and compositions of the EPSG hydrogels are listed in Table 1. For example, EPSG-30 means the ECH-crosslinked PVA/SPI composite hydrogel with 30 wt% of the original PVA weight content.

**TABLE 1:** Codes of the PVA/SPI composite (EPSG) hydrogels with different weight ratios.

Code	PVA (wt%)	SPI (wt%)
EPSG-0	0	100
EPSG-30	30	70
EPSG-50	50	50
EPSG-70	70	30
EPSG-100	100	0

### 2.3 Characterization

The morphology observations of the freeze-dried EPSG hydrogels were conducted by using scanning electron microscope (SEM, VEGA3) at an accelerating voltage of 20 kV. The EPSG hydrogels sheets were frozen in liquid nitrogen first, and then fractured immediately before freeze-drying. The surfaces and cross-sections the EPSG hydrogels were sputtered with gold before observation. The X-ray diffraction (XRD) patterns for the freeze-dried hydrogels were obtained on an XRD diffractometer (D8-Advance, Bruker, United States) with Cu K_α_ radiation (λ = 0.15406 nm) in the region of 2θ from 4 to 40°. The freeze-dried EPSG hydrogels and PVA raw material were cut into powder, which were then dried with SPI powder in a vacuum oven at 60°C for 48 h before testing. FT-IR spectra were recorded on a FT-IR spectrometer (1600, Perkin–Elmer Co., MA) in the wavelength range from 4000 to 400 cm^−1^. The above dried powder samples were used for the FTIR measurement, which were further treated by the KBr disk method. The water content ratios of the hydrogels could be affected by both the chemical compositions and the gelation procedures. The water content ratios were calculated according to Eq. 1:

Water content ratio (%) = [(We -Wd) / We] × 100 (1)

Where W_e_ is the weight of the hydrogels at equilibrium state in water and W_d_ is the weight of the hydrogel at the dry state. For water uptake test, the completely dried EPSG hydrogels (namely xerogels) were weighed (W_0_). The EPSG samples were weighed (W_t_) after soaking in distilled water for an expected time. The values of water uptake ratio for EPSG xerogels were then calculated according to Eq. 2:

Water uptake ratio (%) = [(Wt -W0) / W0] × 100 (2)

The physical mechanical properties of the EPSG hydrogels were measured on a universal tester (CMT 6503, Shenzhen SANS Test machine Co. Ltd.).

### 2.4 Cytocompatibility evaluation of the EPSG-n hydrogels

The cytocompatibility of the EPSG-n hydrogels was evaluated by MTT and direct cell culture methods systematically, which is very important for their application.

#### 2.4.1 Pretreatment of EPSG-n and preparation of the corresponding extracts

The EPSG-n hydrogels were vacuum-dried completely, 1g dried samples were packed with tin foil and sterilized totally in an autoclave before use. The sterilized EPSG-n hydrogels were cut into powder and then immersed in 10 ml MEM 1640 cell culture medium (0.1 g hydrogel/mL). The above solutions were kept in constant temperature incubator at 37 °C for 72h, the resultant supernatants were the corresponding extracts.

#### 2.4.2 MTT assay and direct contact method for cytocompatibility evaluation

MTT assay and direct contact method were used for cytocompatibility evaluation, and the experiment processes were like our previous work (Zhao Y et al., 2018). Briefly, the suspensions with the cell concentration of 1×10^4^ cells/mL were prepared, which were inoculated into 96 well culture plates (100 μL/well). The complete medium with cells was coded as the negative control, and single complete medium was coded as the blank control (for calibration), respectively. After incubation in a CO_2_ Incubator at 37 °C for 24 h, the original culture solution was discarded and PBS was added for rinsing. 150 μL PBS culture solutions and 50 μL EPSG-n extract solutions were added, while the negative control was added with 200 μL medium. The cells were treated with 5 mg/ml MTT after incubating for 24, 48 and 72h, respectively, which were further incubated for 4–5 h. Then, DMSO was added (150 μL/well) after the complete removal of the culture solutions. Absorbance values were read at a test wavelength of 570 nm. The corresponding cell viability for all EPSG hydrogels was calculated using Eq. 3:

$$\text{Cell viability }\left(\text{\%}\right)\;=\;\left({\text{A}}_{\text{test}}/{\text{A}}_{\text{control}}\right)\text{×}100$$ (3)

where A_test_ and A_control_ are the absorption values for the EPSG hydrogels and negative control groups, respectively.

The EPSG-n hydrogels were cut into thin disks with the diameter of 1cm, which were totally before use. The sterilized disks were put in a 6-well cell culture plate, which was treated by ultraviolet radiation for 0.5 h. The resultant samples were then rinsed with PBS solutions for 3 times, the first two rinsings were subjected to ultraviolet irradiation for 0.5h, and the last rinsing was then placed in an incubator. Then, PBS was sucked out the wells and the medium was added in the wells, and the 6-well cell culture plate was kept in the incubator. The treated samples were removed and kept in the wells of a new 24-well culture plate. 100 μL cell suspensions were added onto the surface of EPSG-n and in the blank well (negative control), respectively. The culture plates were taken out after 2 h incubation with the following addition of 900 μl RPMI1640 culture medium. Then, the culture plates were put into the incubator for the following incubation of 72 h. The cells on the above EPSG-n and the negative control were rinsed with PBS solution twice and fixed with 2.5 wt% glutaraldehyde at 4°C for 4 h. The fixed samples were freeze-dried and coated with gold for SEM observation.

### 2.5 Hemocompatibility evaluations of the EPSG-n hydrogels

The effects of the EPSG-n hydrogels on the blood coagulation time and components of blood should be tested systematically to evaluate their potentials as implants. The experimental processes were like our previous work (Zhao Y et al., 2018). Briefly, 2 ml the fresh anticoagulant whole blood (containing 0.38% sodium citrate) was added into 2.5 ml physiological saline, the resultant diluted blood was used for the hemolysis test. The EPSG-n hydrogels with the size of 1 × 1 mm were put into the tubes and rinsed with distilled water for three times, which was further rinsed with 0.9% NaCl solution for 0.5 h and replaced with fresh NaCl solution for another 0.5 h. 0.2 ml above diluted whole blood was added into the above tubes. Then, the resultant solutions after 1 h contact at 37°C were centrifuged for 10 min, and the absorbance of the supernatants after centrifugation was measured. 0.2 ml fresh whole blood was mixed with distilled water to prepare the positive control. 0.2 ml fresh whole blood was also mixed with 0.9 wt% NaCl solution to prepare the negative control. The values of hemolysis ratio (HR) for all the EHSP hydrogels were calculated by Eq. 4:

HR (%) = (AS-AN) / (AP -AN) ×100 (4)

Where AS, AN and AP are the average absorbance of the EPSG-n hydrogels, negative controls and positive controls by measuring three times for each sample, respectively.

Tests of prothrombin time (PT), activated partial thrombin time (APTT) and thrombin time (TT) of the EPSG-n hydrogels were conducted systematically. The hydrogel blocks with the size of 0.2 × 0.2 cm were placed in 96 well plates. Fresh venous blood was mixed homogenously with 0.109M sodium citrate in the proportion of 9:1, which was centrifuged at 3000 rpm for 15 min, the upper plasma was then collected. The plasma was added into wells with the EPSG-n hydrogels, each well was added with 100 μL plasma, and four blank controls were set without materials. The plates were incubated in a water bath pot for 3 min, and then 50 μL pretreated TT reagent (equilibrium at room temperature) was added and blended immediately, the plasma coagulation time was recorded as TT. 0.2 × 0.2 cm hydrogels were put into 96-well plates, and the 50 μl of the above plasma was added into each well with the EPSG-n hydrogels, four blank controls were set without hydrogels. The incubation process was conducted by keeping the plates at 37°C for 3 min. PT reagent was preheated to 37°C, which was then added in the wells of the above plate (100 μL per well) and mixed immediately to measure the plasma coagulation time (PT). Similarly, the above plasma (50 μl) and preheated APTT reagent (50 μl, 37°C) were added into each well containing 0.2 × 0.2 cm EPSG-n hydrogel blocks, the wells without hydrogels was set as the blank control. After the incubation for 3 min, preheated CaCl_2_ reagent (50 μl, 37°C) was added and mixed immediately in each well to measure the plasma coagulation time (APTT).

## 3 Results and Discussion

### 3.1 Appearance and structure of the EPSG-n hydrogels

**FIGURE 1:**
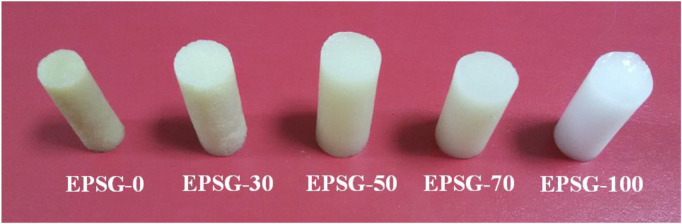
Photographs of the EPSG-n hydrogels (*n* = 0, 30, 50, 70 and 100).

Figure 1 shows the photographs of the EPSG-n hydrogels. Obviously, all the hydrogels exhibited relative homogenous appearances, indicating the successful preparation process for the pure SPI (EPSG-0) and PVA hydrogels (EPSG-100) as well as the composite hydrogels. The EPSG-100 hydrogel showed white color due to the formation of PVA crystallites from the freezing-thawing process (He et al., 2014), and the EPSG-0 hydrogel showed light brown color. Moreover, the color changed gradually from dark to light with the increase of PVA content for the composite hydrogels.

**FIGURE 2:**
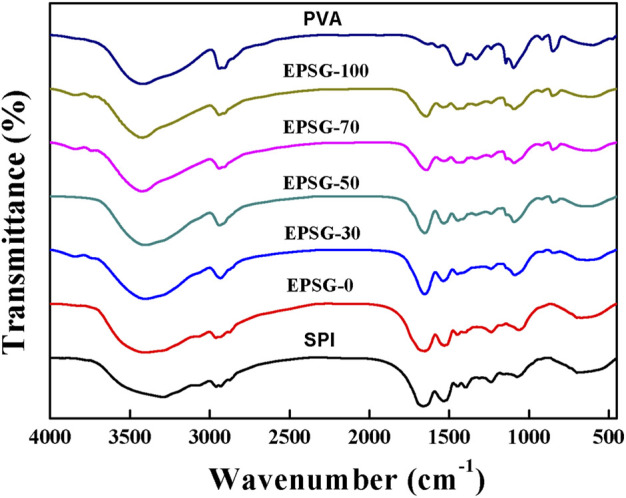
FTIR spectra of the EPSG-n hydrogels (*n* = 0, 30, 50, 70 and 100), and raw materials of PVA and SPI.

Figure 2 (SPI) shows the FTIR spectrum of the original SPI powder. The absorption band at 3292 cm^−1^ was attributed to hydrogen bonding between protein chains and bound water in the protein (Figure 2, SPI). There were obvious -NH bands at 1608–1706 and 1506–1571 cm^−1^, which were in accordance with the amide I and amide II bands for soy protein, respectively (Zhao Y et al., 2016; Schmidt and Soldi, 2006). The absorption band at 1228–1455 cm^−1^ was assigned to the C-N stretching and N-H bending (amide I) vibrations. The band at 1075 cm^−1^ was attributed to the vibrations from out-of-plane C–H bending in an aromatic structure etc (Rhim, et al., 2006). Obviously, the -NH bands for SPI weakened for the ESPG-0 in Figure 2, indicating that the –NH groups from SPI reacted with epoxy groups from ECH and ECH could be used as a chemical crosslinker to fabricate SPI chemical hydrogels. Moreover, the absorption group at 3292 cm^−1^ shifted to 3400 cm^−1^ for EPSG-0, further indicating the occurrence of the chemical crosslinking reaction with ECH. The absorption band at 3400 cm^−1^ for EPSG-0 shifted gradually to 3410 cm^−1^ for EPSG-70 with the increase of PVA content, indicating strong interaction existed between SPI and PVA.

**FIGURE 3:**
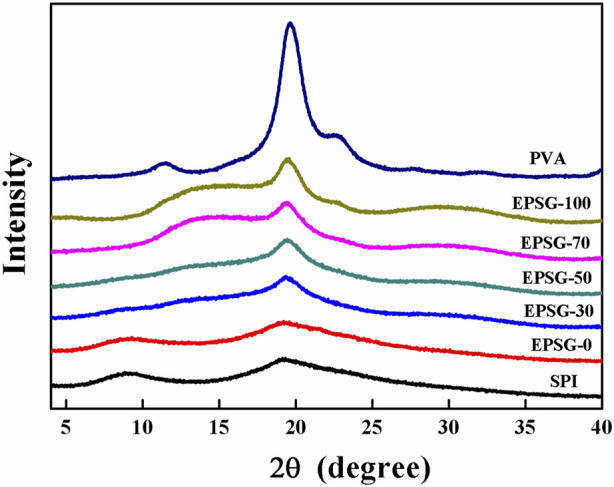
XRD patterns of the EPSG-n hydrogels (*n* = 0, 30, 50, 70 and 100), and raw materials of PVA and SPI.

Figure 3 shows the XRD patterns of EPSG-n (0, 30, 50, and 70), SPI and PVA raw materials. Two peaks at 2θ = 9.0 and 19.2° appeared in the SPI powder, suggesting that there was some ordered structure due to the a-helical structure of the SPI molecules. The peak intensity at 9.0° decreased in EPSG-0, further indicating the chemical crosslinking reaction occurred (Tian K et al., 2010). Moreover, this peak nearly disappeared in the patterns of EPSG-30, EPSG-50 and EPSG-70, indicating that such ordered structure was possibly destroyed by the crosslinking reaction with PVA. PVA had three distinctive diffraction peaks at11.5°, 19.6° and 22.6°, which were assigned to the crystal planes of (100), (101), and (200) planes for PVA (Xu et al., 2015), respectively. Interestingly, the peak at 19.6° for PVA shifted to 19.5° for EPSG-100 with the decrease of intensity, and new broad peaks at 12–17° appeared for EPSG-100 and EPSG-70 possibly due to the crosslinked PVA chains by ECH and their interaction with PVA crystallites, which could destroy the regularity of the original PVA molecules and affect its crystal structure (Chen et al., 2018; Meng et al., 2019). Moreover, the PVA crystal peak still existed in the EPSG-30, EPSG-50 and EPSG-70 hydrogels, suggesting the existence of PVA crystallites (He et al., 2014).

**FIGURE 4:**
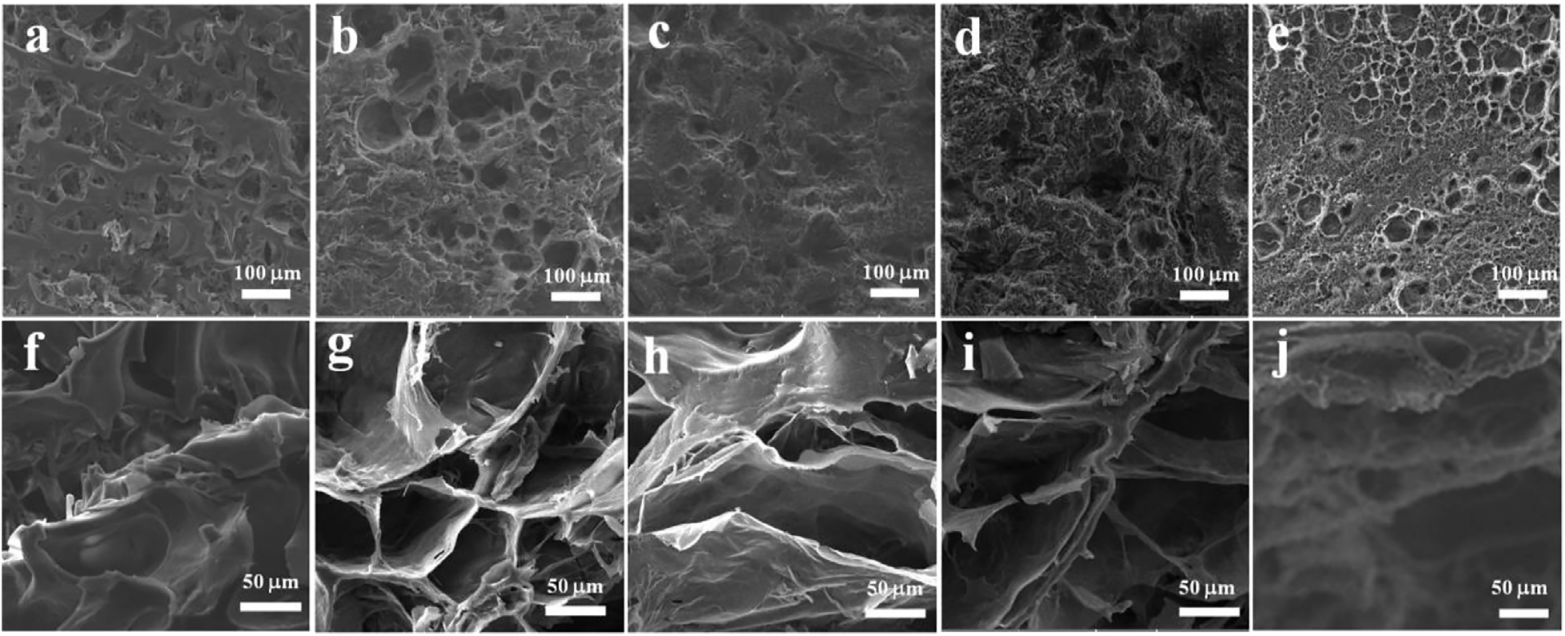
SEM images of the surface **(A–E)** and cross-section **(F–J)** of the EPSG hydrogels for EPSG-0 **(A,F)**, EPSG-30 **(B,G)**, EPSG-50 **(C,H)**, EPSG-70 **(D,I)** and EPSG-100 **(E,J)**, respectively.

The effect of the introduction of PVA on the morphologies for the surface and cross-section of the SPI hydrogel (EPSG-0) was studied by SEM (Figure 4). Obviously, all the hydrogels exhibited relatively homogenous porous structure on the surface (Figure 4 a-e), indicating relatively good miscibility between PVA and SPI. Moreover, porous structure also appeared for all the cross-section of the EPSG-n hydrogels (n = 0, 30, 50, 70 and 100) due to good hydrophilicity of SPI and PVA (Figure 4 f-j) and chemical crosslinking effect.

### 3.2 Physical properties of the EPSG-n hydrogels

**FIGURE 5:**
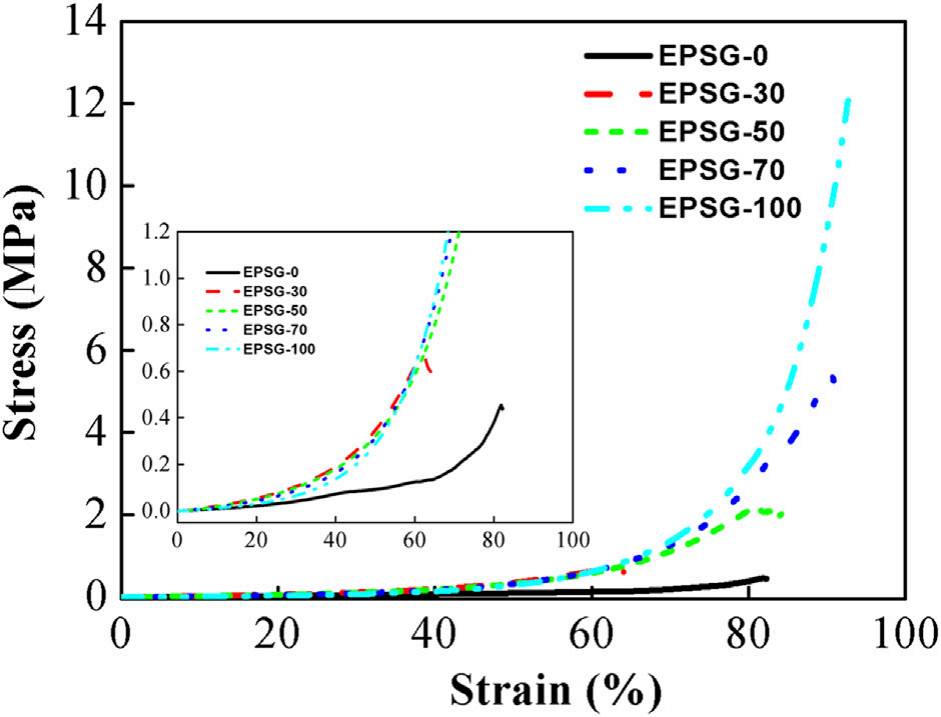
Compressive stress-strain curves of the EPSG-n hydrogels (*n* = 0, 30, 50, 70 and 100).

The mechanical properties are very important for the application of hydrogels in the biomedical field, especially as the supporting biomaterials (such as bone repair materials). The compressive strength for EPSG-0 was 0.45 MPa (Figure 5), which couldn’t satisfy the requirement as some supporting biomaterials. The compressive strength of EPSG-100 could reach 12.3 MPa, which was possibly due to the bi-crosslinking effect from both chemical crosslinking effect through ECH and physical crosslinking effect through PVA crystallites from the freeze-thawing process (He et al., 2014; Ai et al., 2021). The compressive strength increased sharply from 0.45 MPa to 5.38 MPa for EPSG-70 with the increase of PVA content, indicating the successful reinforcement of SPI hydrogels by the introduction of PVA. The reinforcement mechanism for SPI hydrogels by PVA could be possibly due to the formation of a new crosslinking structure between PVA and SPI, and the resultant PVA crystallites from the freeze-thawing process could serve as a “network” in the hydrogel matrix to disperse stress effectively (He et al., 2014).

**FIGURE 6:**
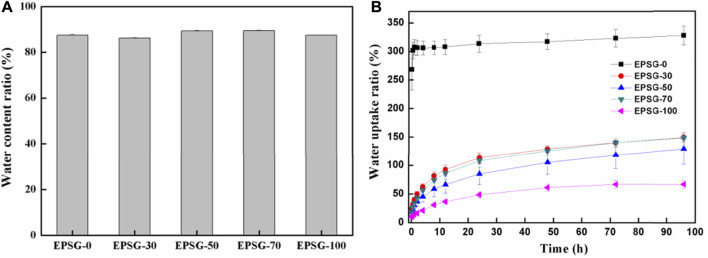
Water content ratios of the EPSG-n hydrogels **(A)** and the water uptake ratios of the completely dried EPSG-n hydrogels **(B)** (n = 0, 30, 50, 70 and 100).

The water content ratios of hydrogels could reflect their hydrophility and micro-structure to some extent. Figure 6A shows the water content ratios of the EPSG-n hydrogels. The water content ratios of all the hydrogels were in the range of 86–90%, showing relatively high-water content due to the good hydrophility of SPI and PVA and chemical crosslinking effect. Figure 6B shows the water uptake ratios of the completely dried EPSG-n hydrogels (xerogels). The EPSG-0 xerogel could absorb water quickly and reach phased equilibrium of 307.6% at 1 h. Compared with EPSG-0, the water absorption ratio of EPSG-100 decreased greatly, which could reach phased equilibrium of 61.1% at 24 h possibly due to the physical crosslinking effect of PVA by forming PVA crystallites through the freeze-thawing process. The water uptake ratio decreased gradually to 327.4% for EPSG-0 and 148.1% for EPSG-70 at 96 h with the incorporation of PVA, indicating that the addition of PVA decreased the ability of SPI protein molecules to absorb and accept water (Su et al., 2010). So, the water uptake ratios of dried EPSG hydrogels could be regulated by controlling PVA contents for different applications. Especially, the decrease of water uptake ratio of the corresponding xerogels could be better for their applications as implants.

### 3.3 Hemocompatibility and cytocompatibility of the EPSG-n hydrogels

As we all know, the hemolysis rate <5% is essential for materials to be used as blood-contacted implants. The hemolysis rates of the groups of positive and negative controls, and the EPSG-n hydrogels (n = 0, 30, 50, 70 and 100) are shown in Table 2, respectively. Obviously, the hemolysis rates of the EPSG-n hydrogels were below 3.5%, showing no hemolysis and little destruction to red blood cells. Thus, the EPSG-n hydrogels could satisfy the requirement of direct blood-contacted biomaterials.

**TABLE 2:** Hemolysis rates of the EPSG-n hydrogels (n = 0, 30, 50, 70 and 100) and the controls.

Sample	HR (%)
Normal saline (Negative control)	100
Distilled water (Positive control)	0
EPSG-0	3.33 ± 0.56
EPSG-30	3.44 ± 1.42
EPSG-50	2.05 ± 0.81
EPSG-70	2.67 ± 0.52
EPSG-100	1.42 ± 0.88

**FIGURE 7:**
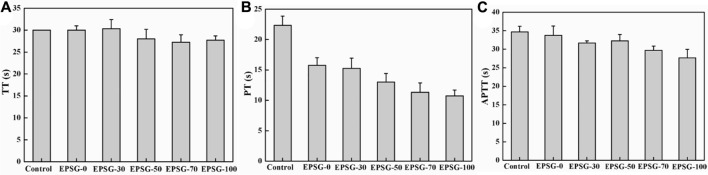
Coagulation routine of the EPSG-n hydrogels (*n* = 0, 30, 50, 70 and 100), TT **(A)**, PT**(B)** and APTT **(C)**.

Moreover, coagulation routine inspection of all the EPSG-n hydrogels (n = 0, 30, 50, 70 and 100) hydrogels was further conducted to evaluate their potentials as implants. TT could reflect the conversion time from fibrinogen to fibrin. Figure 7A shows the TT of EPSG-n (n = 0, 30, 50, 70 and 100). Obviously, the TT values of all the EPSG-n hydrogels were close to the control group, indicating no clinical significance (Zhao et al., 2018). PT could reflect mainly the status of exogenous coagulation system, which could also be used to verify the congenital or acquired fibrin prothrombin and the loss of blood coagulation factorsⅤ, VII and Ⅹor the presence of inhibitor. Figure 7B shows the PT values of EPSG-n (n = 0, 30, 50, 70 and 100). Obviously, the PT values of all the EPSG-n hydrogels were much smaller than that of the blank control, which decreased gradually with the increase of PVA content for all the composite hydrogels, indicating that the hydrogels reduce the coagulation time. Moreover, the status of endogenous blood coagulation system to understand the blood coagulation factors concentrations and the blood coagulation condition could be evaluated by APTT. The APTT values of all the EPSG-n hydrogels were close to the blank control group and in the normal range (Figure 7C), indicating almost no obvious influence on the endogenous clotting of blood.

**FIGURE 8:**
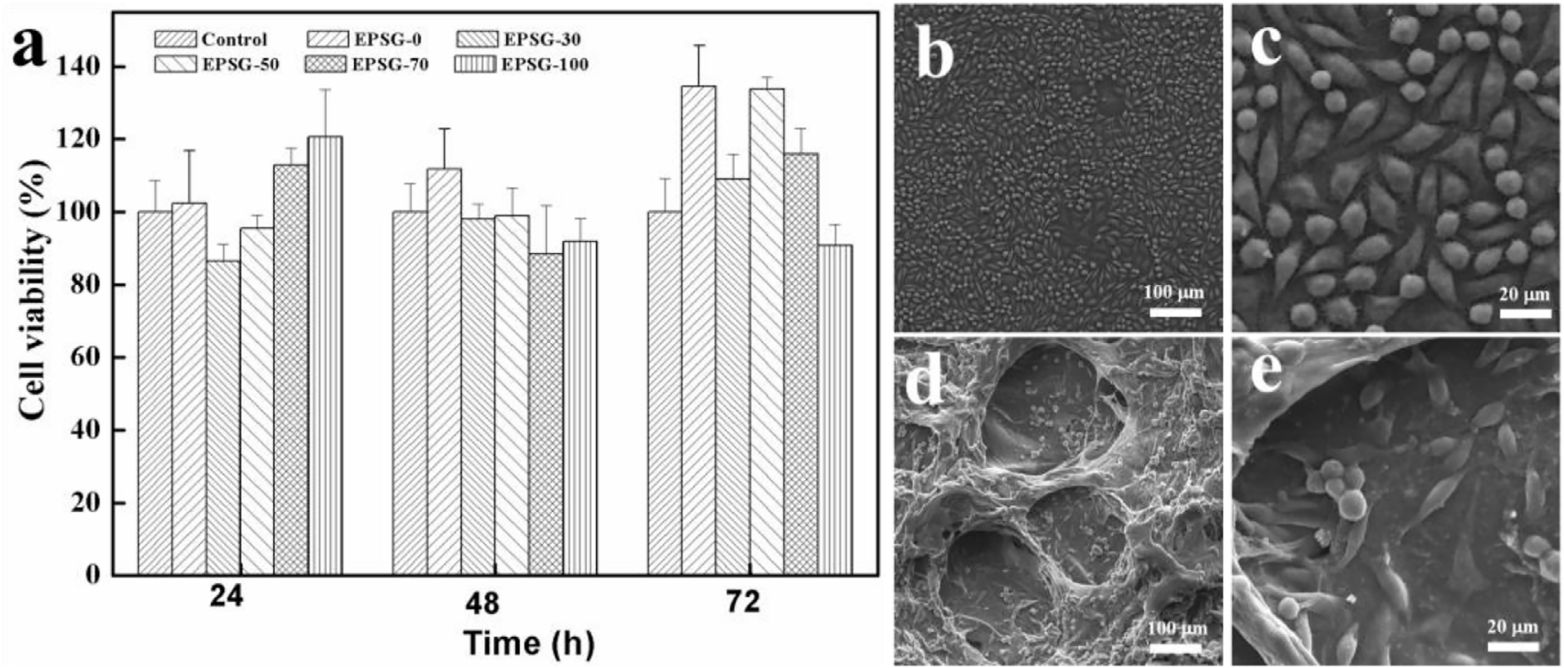
Cell viability of L929 cultured in extracts from EPSG-n hydrogels (*n* = 0, 30, 50, 70 and 100) for 24, 48 and 72 h **(A)**, and SEM images of L929 cells cultured on the control **(B,C)** and the EPSG-50 **(D,E)** hydrogel for 48 h.

The effect of the incorporation of PVA on the cytotoxicity of SPI hydrogels and the corresponding cell viability of L929 for EPSG-n (n = 0, 30, 50, 70 and 100) was evaluated by MTT assay. Figure 8A shows the results of the cytotoxicity tests for EPSG-n. Obviously, all the cell viability values of the EPSG-n hydrogels were above 86.7% at different time of 24, 48 and 72h, showing nearly no cytotoxicity. Interestingly, the cell viability values for EPSG-0 and EPSG-50 at 72 h were a little higher than those of the control, showing no cytotoxicity and the improvement effect on cell proliferation. The adhesion, proliferation and distribution of L929 cells on the control and the EPSG-50 hydrogel after 48 h direct contact were observed by SEM, and the corresponding images are shown in Figures 8B–E. Abundant L929 cells with shuttle-like shape distributed homogenously both on the surface and internal of the EPSG-50 hydrogel, indicating good cytocompatibility.

## 4 Conclusion

High strength EPSG composite hydrogels were constructed successfully by using PVA and SPI as raw materials through the crosslinking with ECH and the freezing-thawing process. There were chemical crosslinking interactions occurred for SPI and PVA during the fabrication process. The composite hydrogels exhibited a homogenous structure and the introduction of PVA increased the compressive strength of SPI hydrogels greatly. Notably, the compressive strength of EPSG-70 could reach 5.38 MPa with the water content ratio of 89.5%. Moreover, the water uptake ratio of the corresponding xerogels decreased gradually from 327.4% for EPSG-0–148.1% for EPSG-70 at 96h, showing a better potential as implants. The cell viability values of all the EPSG hydrogels were above 86.7% at 24, 48 and 72h, showing no cytotoxicity. Moreover, EPSG hydrogels exhibited good biocompatibility and hemocompatibility, showing potential applications as a direct blood contact material in the field of tissue engineering.

## Data Availability

The raw data supporting the conclusion of this article will be made available by the authors, without undue reservation.
